# Bis(2,2-dinitro­prop­yl)formal

**DOI:** 10.1107/S1600536809010642

**Published:** 2009-03-28

**Authors:** Fei Liu, Huajun Dai, Zhong Huang, Yonggang Liu, Xingming Kou

**Affiliations:** a College of Chemistry, Sichuan University, Chengdu 610064, People’s Republic of China; b China Academy of Engineering Physics, Mianyang 621900, People’s Republic of China

## Abstract

The complete mol­ecule of the title compound [systematic name: bis(2,2-dinitro­prop­oxy)methane], C_7_H_12_N_4_O_10_, which was synthesized by the condensation reaction between 2,2-dinitro­propanol and paraformaldehyde in methyl­ene chloride, is generated by crystallographic twofold symmetry with one C atom lying on the rotation axis. In the crystal structure, mol­ecules are linked into chains running parallel to the *b* axis by inter­molecular C—H⋯O hydrogen-bond inter­actions, generating rings of graph-set motif *R*_2_^2^(14).

## Related literature

For the applications and chemistry of the title compound, see: Garver *et al.* (1985 ▶); Hamilton & Wardle (1995 ▶); Adolph (1991 ▶); Hamilton & Wardle (1997 ▶). For graph-set motifs, see: Bernstein (1995 ▶).
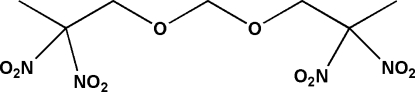


## Experimental

### 

#### Crystal data

C_7_H_12_N_4_O_10_
*M* *_r_* = 312.21
Monoclinic, 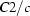

*a* = 23.330 (3) Å
*b* = 6.207 (3) Å
*c* = 10.009 (6) Å
β = 109.60 (3)°
*V* = 1365.6 (11) Å^3^
*Z* = 4
Mo *K*α radiation
μ = 0.14 mm^−1^
*T* = 291 K
0.48 × 0.44 × 0.28 mm

#### Data collection

Enraf–Nonius CAD-4 diffractometer
Absorption correction: none
1404 measured reflections
1255 independent reflections
863 reflections with *I* > 2σ(*I*)
*R*_int_ = 0.008
3 standard reflections every 100 reflections intensity decay: 1.5%

#### Refinement

*R*[*F* ^2^ > 2σ(*F* ^2^)] = 0.046
*wR*(*F* ^2^) = 0.138
*S* = 1.09
1255 reflections
98 parameters
H-atom parameters constrained
Δρ_max_ = 0.18 e Å^−3^
Δρ_min_ = −0.17 e Å^−3^

### 

Data collection: *DIFRAC* (Gabe & White, 1993 ▶); cell refinement: *DIFRAC*; data reduction: *NRCVAX* (Gabe *et al.*, 1989 ▶); program(s) used to solve structure: *SHELXS97* (Sheldrick, 2008 ▶); program(s) used to refine structure: *SHELXL97* (Sheldrick, 2008 ▶); molecular graphics: *ORTEP-3* (Farrugia, 1997 ▶); software used to prepare material for publication: *SHELXL97*.

## Supplementary Material

Crystal structure: contains datablocks global, I. DOI: 10.1107/S1600536809010642/rz2302sup1.cif

Structure factors: contains datablocks I. DOI: 10.1107/S1600536809010642/rz2302Isup2.hkl

Additional supplementary materials:  crystallographic information; 3D view; checkCIF report

## Figures and Tables

**Table 1:** Hydrogen-bond geometry (Å, °)

*D*—H⋯*A*	*D*—H	H⋯*A*	*D*⋯*A*	*D*—H⋯*A*
C1—H1*A*⋯O4^i^	0.97	2.59	3.509 (3)	158
C1—H1*B*⋯O4^ii^	0.97	2.59	3.509 (3)	158

Symmetry codes: (i) 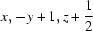; (ii) 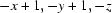.

**Fig. 1.**
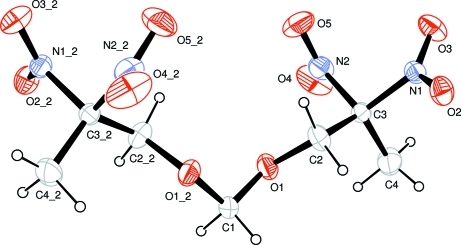
The molecular structure of the title compound, with displacement ellipsoids drawn at the 30% probability level. Atoms labelled with the suffix 2 are generated by the symmetry operator (1-x, y, 1/2-z).

## supplementary crystallographic information

### Comment

The title compound is an important energetic material used in propellant and explosive formulations (Garver *et al.* 1985; Hamilton & Wardle, 1995). It was also combined with liquid bis(2,2-dinitropropyl)acetal (BDNPA) to prepare the BDNPF/A energetic plasticizer (Adolph, 1991; Hamilton & Wardle, 1997). Here we report the crystal structure of the title compound.

The molecule of the title compound (Fig. 1), has crystallographically imposed two-fold symmetry. The average of N—O bond length is 1.204 (3) Å. The dihedral angle formed by the planes through the nitro group is 74.3 (2)°. The O(1)—C(2)—C(3)—N(1), O(5)—N(2)—C(3)—C(4) and O(4)—N(2)—C(3)—C(4) torsion angles are 172.59 (17), -176.2 (2) and 5.6 (3) ° respectively. In the crystal structure, the molecules are linked into chains running parallel to the *b* axis by intermolecular C—H···O hydrogen interactions (Table 1) generating rings of graph set motif *R*^2^_2_(14).

### Experimental

The title compound was synthesized by reacting 2,2-dinitropropanol (6.0 g) with paraformaldehyde (0.6 g) in the presence of concentrated sulfuric acid as catalyst in methylene chloride below 5°C. Single crystals suitable for X-ray analysis were obtained by slow evaporation of a diethyl ether/mineral ether (1:6 v/v) solution.

### Refinement

All non-H atoms were refined with anisotropic atomic displacement parameters. All H atoms were positioned geometrically and refined using a riding model, with C—H =0.96–0.97 Å and with U_iso_(H) = 1.2 U_eq_(C) or 1.5 U_eq_(C) for methyl H atoms.

### Crystal data

**Table:**

C_7_H_12_N_4_O_10_	*F*(000) = 648
*M**_r_* = 312.21	*D*_x_ = 1.519 Mg m^−^^3^
Monoclinic, *C*2/*c*	Mo *K*α radiation, λ = 0.71073 Å
Hall symbol: -C 2yc	Cell parameters from 23 reflections
*a* = 23.330 (3) Å	θ = 5.2–8.7°
*b* = 6.207 (3) Å	µ = 0.14 mm^−^^1^
*c* = 10.009 (6) Å	*T* = 291 K
β = 109.60 (3)°	Block, colourless
*V* = 1365.6 (11) Å^3^	0.48 × 0.44 × 0.28 mm
*Z* = 4	

### Data collection

**Table:**

Enraf–Nonius CAD-4 diffractometer	*R*_int_ = 0.008
Radiation source: fine-focus sealed tube	θ_max_ = 25.5°, θ_min_ = 1.9°
graphite	*h* = −28→20
ω/2θ scans	*k* = 0→7
1404 measured reflections	*l* = −12→12
1255 independent reflections	3 standard reflections every 100 reflections
863 reflections with *I* > 2σ(*I*)	intensity decay: 1.5%

### Refinement

**Table:**

Refinement on *F*^2^	Secondary atom site location: difference Fourier map
Least-squares matrix: full	Hydrogen site location: inferred from neighbouring sites
*R*[*F*^2^ > 2σ(*F*^2^)] = 0.046	H-atom parameters constrained
*wR*(*F*^2^) = 0.138	*w* = 1/[σ^2^(*F*_o_^2^) + (0.0654*P*)^2^ + 0.7176*P*] where *P* = (*F*_o_^2^ + 2*F*_c_^2^)/3
*S* = 1.09	(Δ/σ)_max_ < 0.001
1255 reflections	Δρ_max_ = 0.18 e Å^−^^3^
98 parameters	Δρ_min_ = −0.17 e Å^−^^3^
0 restraints	Extinction correction: *SHELXL97* (Sheldrick, 2008), Fc^*^=kFc[1+0.001xFc^2^λ^3^/sin(2θ)]^-1/4^
Primary atom site location: structure-invariant direct methods	Extinction coefficient: 0.032 (3)

### Special details

**Table:**

Geometry. All e.s.d.'s (except the e.s.d. in the dihedral angle between two l.s. planes) are estimated using the full covariance matrix. The cell e.s.d.'s are taken into account individually in the estimation of e.s.d.'s in distances, angles and torsion angles; correlations between e.s.d.'s in cell parameters are only used when they are defined by crystal symmetry. An approximate (isotropic) treatment of cell e.s.d.'s is used for estimating e.s.d.'s involving l.s. planes.
Refinement. Refinement of *F*^2^ against ALL reflections. The weighted *R*-factor *wR* and goodness of fit *S* are based on *F*^2^, conventional *R*-factors *R* are based on *F*, with *F* set to zero for negative *F*^2^. The threshold expression of *F*^2^ > σ(*F*^2^) is used only for calculating *R*-factors(gt) *etc*. and is not relevant to the choice of reflections for refinement. *R*-factors based on *F*^2^ are statistically about twice as large as those based on *F*, and *R*- factors based on ALL data will be even larger.

### Fractional atomic coordinates and isotropic or equivalent isotropic displacement parameters (Å^2^)

**Table:**

	*x*	*y*	*z*	*U*_iso_*/*U*_eq_	Occ. (<1)
O1	0.54239 (7)	0.5416 (3)	0.21472 (18)	0.0661 (6)	
O2	0.68680 (8)	0.8202 (3)	0.51558 (17)	0.0706 (6)	
O3	0.70016 (9)	1.0217 (3)	0.3547 (2)	0.0854 (7)	
O4	0.60961 (13)	0.8225 (4)	0.0432 (2)	0.1153 (10)	
O5	0.57606 (12)	1.0244 (4)	0.1735 (3)	0.1154 (9)	
N1	0.67637 (8)	0.8730 (3)	0.3932 (2)	0.0538 (5)	
N2	0.60412 (11)	0.8729 (4)	0.1542 (2)	0.0702 (7)	
C1	0.5000	0.4161 (6)	0.2500	0.0721 (11)	
H1A	0.5211	0.3241	0.3297	0.087*	0.50
H1B	0.4789	0.3241	0.1703	0.087*	0.50
C2	0.58346 (10)	0.6513 (4)	0.3320 (2)	0.0619 (7)	
H2A	0.5991	0.5550	0.4124	0.074*	
H2B	0.5636	0.7721	0.3596	0.074*	
C3	0.63384 (9)	0.7279 (3)	0.2821 (2)	0.0470 (6)	
C4	0.67114 (13)	0.5524 (5)	0.2509 (4)	0.0887 (10)	
H4A	0.7000	0.6135	0.2122	0.133*	
H4B	0.6450	0.4542	0.1835	0.133*	
H4C	0.6924	0.4765	0.3368	0.133*	

### Atomic displacement parameters (Å^2^)

**Table:**

	*U*^11^	*U*^22^	*U*^33^	*U*^12^	*U*^13^	*U*^23^
O1	0.0497 (9)	0.0705 (11)	0.0702 (11)	−0.0102 (8)	0.0098 (8)	−0.0145 (8)
O2	0.0670 (11)	0.0859 (12)	0.0506 (10)	−0.0086 (9)	0.0086 (7)	−0.0062 (9)
O3	0.0912 (13)	0.0750 (13)	0.0901 (14)	−0.0353 (11)	0.0306 (11)	−0.0070 (10)
O4	0.179 (3)	0.1115 (18)	0.0553 (12)	−0.0540 (17)	0.0396 (14)	−0.0049 (12)
O5	0.1221 (19)	0.0748 (14)	0.127 (2)	0.0347 (14)	0.0122 (15)	0.0233 (13)
N1	0.0481 (10)	0.0533 (11)	0.0610 (12)	−0.0045 (9)	0.0193 (9)	−0.0076 (9)
N2	0.0834 (15)	0.0650 (14)	0.0552 (13)	−0.0133 (12)	0.0137 (11)	0.0046 (11)
C1	0.0401 (16)	0.0499 (18)	0.114 (3)	0.000	0.0094 (17)	0.000
C2	0.0580 (13)	0.0718 (15)	0.0523 (13)	−0.0174 (12)	0.0138 (10)	−0.0065 (11)
C3	0.0464 (11)	0.0457 (11)	0.0454 (11)	0.0006 (9)	0.0107 (9)	−0.0038 (9)
C4	0.0676 (16)	0.0807 (19)	0.108 (2)	0.0146 (14)	0.0159 (15)	−0.0383 (18)

### Geometric parameters (Å, °)

**Table:**

O1—C1	1.394 (3)	C1—H1A	0.9700
O1—C2	1.416 (3)	C1—H1B	0.9700
O2—N1	1.211 (2)	C2—C3	1.500 (3)
O3—N1	1.205 (2)	C2—H2A	0.9700
O4—N2	1.202 (3)	C2—H2B	0.9700
O5—N2	1.198 (3)	C3—C4	1.491 (3)
N1—C3	1.514 (3)	C4—H4A	0.9600
N2—C3	1.528 (3)	C4—H4B	0.9600
C1—O1^i^	1.394 (3)	C4—H4C	0.9600
			
C1—O1—C2	113.52 (16)	O1—C2—H2B	110.7
O3—N1—O2	124.98 (19)	C3—C2—H2B	110.7
O3—N1—C3	118.63 (19)	H2A—C2—H2B	108.8
O2—N1—C3	116.20 (18)	C4—C3—C2	114.6 (2)
O5—N2—O4	126.0 (3)	C4—C3—N1	107.70 (18)
O5—N2—C3	116.5 (2)	C2—C3—N1	109.75 (17)
O4—N2—C3	117.5 (2)	C4—C3—N2	112.8 (2)
O1—C1—O1^i^	112.1 (3)	C2—C3—N2	106.23 (18)
O1—C1—H1A	109.2	N1—C3—N2	105.44 (18)
O1^i^—C1—H1A	109.2	C3—C4—H4A	109.5
O1—C1—H1B	109.2	C3—C4—H4B	109.5
O1^i^—C1—H1B	109.2	H4A—C4—H4B	109.5
H1A—C1—H1B	107.9	C3—C4—H4C	109.5
O1—C2—C3	105.36 (18)	H4A—C4—H4C	109.5
O1—C2—H2A	110.7	H4B—C4—H4C	109.5
C3—C2—H2A	110.7		
			
C2—O1—C1—O1^i^	70.17 (16)	O3—N1—C3—N2	−31.8 (3)
C1—O1—C2—C3	165.8 (2)	O2—N1—C3—N2	152.91 (19)
O1—C2—C3—C4	−66.1 (3)	O5—N2—C3—C4	−176.2 (2)
O1—C2—C3—N1	172.59 (17)	O4—N2—C3—C4	5.6 (3)
O1—C2—C3—N2	59.1 (2)	O5—N2—C3—C2	57.6 (3)
O3—N1—C3—C4	88.8 (3)	O4—N2—C3—C2	−120.7 (2)
O2—N1—C3—C4	−86.4 (3)	O5—N2—C3—N1	−58.9 (3)
O3—N1—C3—C2	−145.9 (2)	O4—N2—C3—N1	122.9 (2)
O2—N1—C3—C2	38.9 (3)		

Symmetry codes: (i) −*x*+1, *y*, −*z*+1/2.

### Hydrogen-bond geometry (Å, °)

**Table:**

*D*—H···*A*	*D*—H	H···*A*	*D*···*A*	*D*—H···*A*
C1—H1A···O4^ii^	0.97	2.59	3.509 (3)	158
C1—H1B···O4^iii^	0.97	2.59	3.509 (3)	158

Symmetry codes: (ii) *x*, −*y*+1, *z*+1/2; (iii) −*x*+1, −*y*+1, −*z*.
